# Optimal Complexity of Parameterized Quantum Circuits

**DOI:** 10.3390/e28010073

**Published:** 2026-01-08

**Authors:** Guilherme I. Correr, Pedro C. Azado, Diogo O. Soares-Pinto, Gabriel G. Carlo

**Affiliations:** 1 Instituto de Física de São Carlos, Universidade de São Paulo, IFSC–USP, São Carlos 13566-590, SP, Brazil; pedroazado@ifsc.usp.br (P.C.A.); dosp@ifsc.usp.br (D.O.S.-P.); 2 QTF Centre of Excellence, Department of Physics, University of Helsinki, FIN-00014 Helsinki, Finland; 3 International Iberian Nanotechnology Laboratory (INL), 4715-330 Braga, Portugal; 4 Consejo Nacional de Investigaciones Científicas y Técnicas (CONICET), Departamento de Física, Comisión Nacional de Energía Atómica, Av. del Libertador 8250, Buenos Aires 1429, Argentina

**Keywords:** parameterized quantum circuits, expressibility, majorization, entanglement

## Abstract

Parameterized quantum circuits are central to the development of variational quantum algorithms in the NISQ era. A key feature of these circuits is their ability to generate an expressive set of quantum states, enabling the approximation of solutions to diverse problems. The expressibility of such circuits can be assessed by analyzing the ensemble of states produced when their parameters are randomly sampled, a property closely tied to quantum complexity. In this work, we compare different classes of parameterized quantum circuits with a prototypical family of universal random circuits to investigate how rapidly they approach the asymptotic complexity defined by the Haar measure. We find that parameterized circuits exhibit faster convergence in terms of the number of gates required, as quantified through expressibility and majorization-based complexity measures. Moreover, the topology of qubit connections proves crucial, significantly affecting entanglement generation and, consequently, complexity growth. The majorization criterion emerges as a valuable complementary tool, offering distinct insights into the behavior of random state generation in the considered circuit families.

## 1. Introduction

The classification of quantum random circuits according to their complexity has become an active area of research. On the one hand, random quantum circuits are important simulators of quantum dynamics, being of fundamental importance to generate approximations of Haar random unitaries [1] and for the understanding of different kinds of many-body dynamics [2,3,4]. Often, these applications rely on highly complex quantum circuits to achieve specific tasks. On the other hand, it is possible to say that the main reason behind this complexity characterization is that with the advent of the so-called NISQ devices novel platforms have emerged as potential probes to test quantum advantage. Knowing which are the best architectures for implementing different quantum protocols like variational quantum algorithms, is also highly relevant [5,6,7,8]. In this context, parameterized quantum circuits play a central role in the development of efficient quantum algorithms. However, their characteristics are not fully understood, even more when considered outside the scope of variational algorithms. In this sense, the study of their complexity growth is highly interesting to investigate the possibilities of applications these circuits based on NISQ devices can achieve.

There are several measures of complexity for quantum circuits [9,10,11,12]. Some of them are based on a comparison between the uniform and invariant measure over the space of the group, the Haar measure, and the random unitaries generated by the circuit. A recently introduced measure of this kind, the majorization criterion [13], is based on the fluctuations of Lorenz curves. These curves are defined by the cumulants of a given ordered distribution. By comparing these curves with the one obtained considering Haar sampled unitaries, it is possible to characterize the complexity of the circuit. Another measure that has attracted a lot of attention recently is the expressibility [14]. This measure can be translated into the relative entropy comparing the distribution of fidelities of states generated by the random circuit with the distribution of the Haar random case. We have also considered the average entanglement that determines the average and standard deviation values for the entanglement of states generated by the circuits. This later quantity contains important information about the characteristics of the circuit and can be made suitable to future complexity assessments [2,10,15,16,17,18]. For example, random circuits close in behavior to the Haar measure, i.e., close to a *t*-design [19,20,21] or close to the generation of uniformly distributed states, generate average entanglement that approximate well the values obtained by sampling with the Haar measure [15,16,17]. Therefore, it can be seen as a necessary condition and another tool to understand the evolution of entanglement correlations when circuit complexity increases.

In this work we compare these three measures for different configurations of parameterized quantum circuits and an universal class of random circuits generated by few gates and widely used in several implementations of NISQ benchmarking. As a result of our comparison, we could identify that the rate of convergence towards the optimal complexity characterized by Haar-like fluctuations in the Lorenz curves is greater for the parameterized circuits given by the most connected topologies than the non-parametric class. This is consistent with the results obtained using the expressibility and the mean entanglement.

The paper is organized as follows: In Section 2 we explain the construction of the different classes of circuits we analyze. In the following Section 3, we define the measures used to quantify the complexity of the previously defined circuits. Results are shown in Section 4. Finally, we offer the concluding remarks in Section 5.

## 2. Quantum Circuits

The main class of circuits that we are going to analyze are the so called parameterized quantum circuits (PQCs). These are fixed structures of parameterized gates of one and two qubits, concatenated many times to achieve different objectives [14,22]. In the context of variational quantum algorithms (VQAs), these parameters are optimized by applying a classical optimization method together with a cost function that encodes the solution of a particular problem to be solved. Still, a different option to explore the possibilities for PQCs is to sample the parameters at random to obtain circuits generating ensembles of random unitaries or random states [23,24,25], in a very close manner to pseudorandom circuits [1,3,15] (Appendix A).

**Figure 1:**
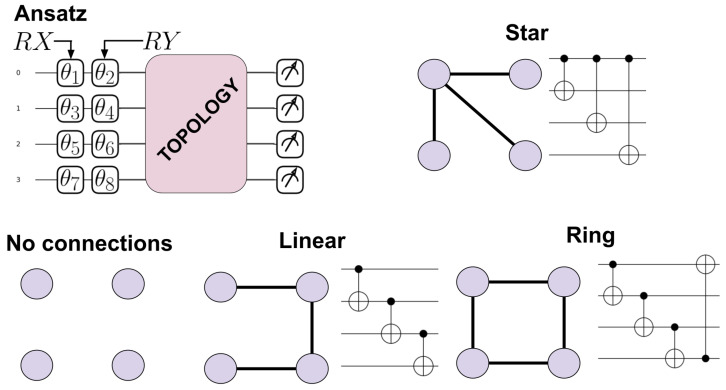
Circuit ansatz (fixed structure that is concatenated) and different topologies of connections considered here for 4 qubits. The rotations part of the circuit is implemented by a set of RX rotations (first vertical set of gates) followed by a set of RY rotations (second vertical set of gates), each with independent sampled angles $\theta_{n}$. The circuits are generated by changing the “TOPOLOGY” part to the CNOT circuit representation of the graphs. In the case of *No connections*, nothing is done in the topology step.

The structures of the PQCs in this work are chosen both to simplify the local parameterized operations and to match the connectivities available in the IBM quantum computers with H topology and others with similar square/rectangular topology [26,27,28]. In the study of quantum computer architectures, the usual nomenclature for the connections that can be performed between qubits is called the topology, connectivity, or architecture of the hardware. In this work and to avoid confusion, here we clearly state that we have chosen the term “topology” to identify the structure of the graph related to a particular connectivity between qubits that a computer can have. We considered 4 to 8 qubits and 1 to 10 circuit concatenations with independent parameters, called number of layers in the context of VQAs [22]. To compare with random circuits composed of discrete gates, the number of layers is translated to number of gates according to Table 1. Figure 1 presents the circuit ansätze, where RX and RY are applied to every qubit with random parameters sampled according to the uniform distribution between 0 and 2π, followed by the connections, represented as graphs and as digital circuits. CNOTs are used as the two qubits gates responsible for the connections. We present only the 4 qubits case as an illustration. The sequence of gates RX and RY with parameters uniformly sampled is not capable of generating uniformly distributed states of one qubit when considering the |0⟩ input state [14]. Still, this choice is capable of obtaining states distributed around the Bloch sphere, and can lead to random distributed states close to the uniform distribution [14,29]. We emphasize that the parameterized circuits considered here are not intended to have optimal depth. Instead, we choose the arrangement of entangling operations according to a particular connectivity graph, allowing us to study complexity growth given the number of gates applied.

The circuits are executed many times with different parameters to generate the states used to calculate the quantifiers. Each of the parameters appearing in the parameter vector are sampled considering independent and identically distributed random variables, representing the uniform distribution in the flat torus.

For comparison purposes we are also going to study the behavior of a standard class of universal quantum circuits that is constructed by means of a few generators and which is a standard model for universal quantum computation. This is given by G3={CNOT,H,T}, where *H* stands for Hadamard and *T* are π/8 phase gates. The set *G3* has been proven to be universal, approximating the unitary group U(N) to desired precision [13,30,31]. To construct these circuits, we take equal probability for each gate at a given time, and also equal probability for the qubits or pairs of qubits to apply them. In this construction, gates are randomly sampled without memory. Therefore, gate cancellations are allowed and not explicitly removed.

Another class of circuits that could have been taken into account is the one given by G2={CNOT,H,S}, also known as the generators of the Clifford group [32]. This class of circuits, however, is classically simulatable [33], which means that they cannot fully exploit the advantages of entanglement and expressiveness generated by a universal set of gates. From previous works [34,35], we have seen that the G3 family generates a more uniformly distributed set of states in the state space than G2 (in fact, it is able to simulate a Haar-generated set given enough gates).

## 3. Complexity Quantifiers

### 3.1. Expressibility

The expressibility is a figure of merit proposed in the context of parameterized quantum circuits to analyze how uniformly distributed the pure states generated by the circuit in the state space are [14]. To do so, the circuit averaged state over randomly distributed parameters is compared to the averaged state considering the uniformly distributed Haar measure [14]

(1) $$A^{\left(t\right)}=\int_{Haar}{\left(|\psi \rangle \langle \psi |\right)}^{⊗t}d\psi -\int_{\Theta }{\left(|\phi (\theta )\rangle \langle \phi (\theta )|\right)}^{⊗t}d\theta ,$$

where Θ is the space of parameter vectors given as input for the circuit, considering a particular distribution for the sampling, and dψ is the uniformly distributed Haar-induced measure over pure states space [36,37]. The Hilbert–Schmidt norm is calculated for this quantity and the closer it is to 0, the closer the circuit is to generating uniformly distributed states. This more rigorous definition compares the circuits with *t*-designs, which is a good measure to quantify how close a circuit is to a particular design order (i.e., how close the moments of the circuit are to the Haar ones up to the *t*-th moment [19,20,38]). However, a more broad and operationally meaningful quantifier based in the same notion is defined by the relative entropy computed considering the distribution of fidelities comparing two states generated by the circuit and the same distribution for Haar random states. The relative entropy or Kullback–Leibler divergence [39,40] is defined as

(2) $$D_{KL}\left(P||Q\right)=\sum_{x}P\left(x\right)\log\left(\frac{P(x)}{Q(x)}\right).$$

The distributions *P* and *Q* are the fidelity distributions. First, the circuit distribution is computed sampling states with different parameters |ψ(θ)⟩, |ψ(φ)⟩ and calculating the fidelity $F\left(\theta ,\varphi \right)={\left|\left\langle \psi (\theta \right)|\psi (\varphi )\rangle \right|}^{2}$. From sampling many different states, a histogram can be built, $P_{PQC}\left(F\right)$. We applied 75 bins in the interval [0,1], which provides a bin size of 0.013. The sample size was $10^{4}$ parameter vectors and, therefore, output states, generating $5\times 10^{3}$ fidelities. In each plot, data points are averaged over $10^{4}$ independent circuit realizations. Error bars representing the standard error of the mean are omitted as they are smaller than the marker size. This histogram is then compared with the one obtained with the probability density function of fidelities for Haar random states, $PDF_{\mathrm{Haar}}\left(F\right)=\left(d-1\right){\left(1-F\right)}^{d-2}$, where *d* is the dimension of the system [41]. We call this histogram $P_{\mathrm{Haar}}\left(F\right)$. This way, to estimate how uniformly distributed the states generated by the circuit are, we compute

(3) $$\mathrm{Expr}:=D_{KL}\left(P_{PQC}\left(F\right)\left|\right|P_{\mathrm{Haar}}\left(F\right)\right).$$

The closer Expr is to zero, the more uniformly distributed the states generated by the circuit in the state space are. Usually, it is then said that the circuit-induced measure is more *expressible*. To avoid misunderstandings, we are going to refer to Expr as $D_{KL}$ or relative entropy, and the closer this quantity is to zero, the higher the expressibility. For more details on the calculations of the histograms and parameters considered, please refer to the authors’ project on github (Available at: https://github.com/GICorrer/Expressibility-and-Scott-entanglement-measure-for-Parameterized-Quantum-Circuits) (accessed on 5 January 2026).

### 3.2. Majorization Criterion

In trying to grasp the complexity of quantum circuits (and devices) a measure inspired by the majorization principle has recently been proposed [13]. Majorization refers to a way of ordering vectors according to the distribution of their components. We can take any two vectors $p,q\in R^{N}$, for example. If

(4) $$\begin{array}{cc} \sum_{i=1}^{k}p_{i}^{↓} & \leq \sum_{i=1}^{k}q_{i}^{↓},\;1\leq k<N, \end{array}$$

(5) $$\begin{array}{cc} \sum_{i=1}^{N}p_{i} & =\sum_{i=1}^{N}q_{i}, \end{array}$$

where ^↓^ stands for sorting the components in non-increasing order, then **p** is majorized by **q**. This is usually written as p≺q. In the context relevant for this manuscript, where the vectors characterize probability distributions (entries are non-negative and sum to one), the majorization indicates that the components of **p** are more uniformly distributed than the components of **q** [42]. In our case the components are the probabilities associated to the output state vectors of a given quantum circuit (normalized). The *k*-th partial sum in Equation (4) is called the *k*-th cumulant of either p or q ($F_{p}\left(k\right)$ and $F_{q}\left(k\right)$, respectively). It is then clear that if p≺q, then $F_{p}\left(k\right)\leq F_{q}\left(k\right)$ for 1≤k<N. The plots of $F_{p}\left(k\right)$ and $F_{q}\left(k\right)$ vs. k/N are the *Lorenz curves* and saying that **q** majorizes **p** is equivalent to the Lorenz curve for **q** being always above the curve for **p**.

If we consider an ensemble of *n*-qubit random quantum circuits {U} of a given class, we can measure this class complexity by studying the fluctuations of the Lorenz curves. This is accomplished by uniformly sampling the corresponding circuits in order to make them act on an initial state given by $|0\ldots 0\rangle ={|0\rangle }^{⊗n}$ and finally measuring in the computational basis. This gives the output distributions, $p_{U}\left(i\right)={\left|\left\langle 0\ldots 0\right|U|i\rangle \right|}^{2}$, whose cumulants $F_{p_{U}}\left(k\right)$–with $k\in \{1,\ldots ,2^{n}\}$ are used to evaluate the fluctuations

(6) $$\begin{array}{c} \mathrm{std}\;\left[F_{p_{U}}\left(k\right)\right]=\sqrt{\langle F_{p_{U}}^{2}\left(k\right)\rangle -{\langle F_{p_{U}}\left(k\right)\rangle }^{2}}. \end{array}$$

The quantum complexity is given by the distance of these fluctuations with respect to the ones that are characteristic of *n*-qubit Haar-random pure states. By computing such distance, we are left with a single quantifier depending on the circuit, given by

(7) $$D_{H}=\sqrt{\sum_{k=1}^{2^{n}}\left\{\mathrm{std}\;\left[F\left(k\right)\right]-\mathrm{std}\;\left[F_{H}\left(k\right)\right]\right\}}.$$

As a matter of fact, the Haar-*n* curve provides a lower limit for universal gate sets [35], being a reference for identifying quantum complexity unreachable by means of classical computations in the large *n* limit. This criterion not only allows us to single out the complexity associated with universal and non-universal classes of random quantum circuits, but also some non-universal but not classically efficiently simulatable ones. Very interesting applications in reservoir quantum computing have recently been reported [34,43].

### 3.3. Average Entanglement

To quantify the entanglement generated by random quantum circuits, we consider the Meyer–Wallach multipartite entanglement measure [44]. This quantity was first defined in terms of the wedge product and later Brennen obtained a decomposition in terms of the linear entropy entanglement quantifier, $S_{L}\left(\sigma_{A}\right)=1-\mathrm{Tr}(\sigma_{A}^{2})$ [45,46], where $\sigma_{AB}$ is a bipartite system with subsystems *A*, *B*, and $\sigma_{A}={\mathrm{Tr}}_{B}\left(\sigma_{AB}\right)$ is the reduced state of system *A*. For a pure state of *n* qubits, |ψ⟩, it is calculated as

(8) $$Q\left(|\psi \rangle \right)=\frac{2}{n}\sum_{k=1}^{n}S_{L}\left(\rho_{k}\right)=2\left[1-\frac{1}{n}\sum_{k=1}^{n}\mathrm{Tr}(\rho_{k}^{2})\right],$$

where $\rho_{k}={\mathrm{Tr}}_{\hat{k}}\left(|\psi \rangle \langle \psi |\right)$ is the reduced state obtained by tracing out all the qubits but the *k*-th. This entanglement quantifier is then based on the mean value of the linear entropy considering every possible bipartition of one qubit/rest of the system. This quantifier will be maximum when the linear entropy of every bipartition is maximum, i.e., equal to 1. In Equation (8), this is the same as every reduced state being maximally mixed.

The fact that it reaches its maximum for states with very different multipartite entanglement constitutes a drawback of such measure, being unable to discern its kind. Taking for example the four qubits’ GHZ state and the tensor product of two Bell states, both would yield entanglement equal to one. Nevertheless, the fact that we can express it as a linear function of the average purity of the qubits [45] makes it an easily computable measure of entanglement, without the need for a full quantum state tomography.

There are generalizations of this measure for systems with different bipartition sizes (e.g., two qubits and the rest of the system) and different subsystem dimensions [17,47,48] that we are not going to consider in this work. Still, the Meyer–Wallach measure is an important entanglement quantifier in the context of pseudorandom circuits and chaotic dynamics, being applied to characterize randomness [15], to diagnose chaotic behavior [17], and to discuss average properties of the entanglement in quantum circuits [14]. In this sense, considering that this measure is well-suited as a generalization of linear entropy for measuring multipartite entanglement, and the history in previous applications with analytical and numerical results [14,15,16,17,29], it was chosen in this work as the most suitable entanglement measure to characterize the circuits.

The circuits will generate very different states depending on the parameter vector given as input. For example, a circuit where all the angles in the parameter vector are 0 and the input state is ${\left|0\right\rangle }^{⊗n}$, *n* number of qubits, will not generate entanglement if only RX, RY and RZ parameterized local operations and CNOT gates are used. This way, to verify the properties of the entanglement generation of the circuit, we perform an average over an ensemble of parameter vectors as

(9) $${\langle Q\rangle }_{\Theta }=\frac{1}{N}\sum_{i=1}^{N}Q\left(\left|\psi (\theta_{i})\right\rangle \right),$$

where $N=10^{4}$ is the sample size and $\theta_{i}$ are different parameter vectors. Each parameter in the parameter vector is sampled according to the uniform distribution between 0 and 2π. We have also computed the standard deviation, using a very similar procedure where the average and the squared value average were calculated to determine the values.

The mean value and standard deviation of this entanglement measure in the case of a Haar-induced uniform measure in the space of pure states were calculated in Refs. [16,17]. They read, for an *n*-qubit Hilbert space,

(10) $$\begin{array}{ccc} {\langle Q\rangle }_{\mathrm{Haar}} & = & \frac{2^{n}-2}{2^{n}+1},\\ \sigma_{\mathrm{Haar}}\left(Q\right) & = & \sqrt{\frac{6(2^{n}-4)}{\left(2^{n}+3\right)\left(2^{n}+2\right)\left(2^{n}+1\right)n}+\frac{18\cdot 2^{n}}{\left(2^{n}+3\right)\left(2^{n}+2\right){\left(2^{n}+1\right)}^{2}}}. \end{array}$$

This relation will be essential to understand the convergence of the entanglement measure to the average value ${\langle Q\rangle }_{\mathrm{Haar}}$ as we increase the circuits’ number of gates. This convergence of the average value is characteristic of random quantum circuits and of circuits that are generating unitary designs of order 2 [14,15,16,17,29]. This way, it works as a necessary condition of convergence to a 2-design or to characterize closeness to the generation of uniformly distributed random states. Notice that the convergence to the value $\sigma_{\mathrm{Haar}}\left(Q_{1}\right)$ is not guaranteed by a 2-design.

### 3.4. Relation to Dimensional Expressivity and Controllability

An important remark we should make about these descriptors we chose is that expressibility measures employed in this work are closely related to, yet conceptually distinct from, dimensional expressivity and controllability results developed in quantum control theory and variational quantum algorithms [14,22,49,50,51,52,53]. Dimensional expressivity characterizes the dimension of the manifold of quantum states or unitaries reachable by a given parameterized quantum circuit as a function of the number of layers and independent parameters, and leads to notions such as pure-state controllability (PSC) and operator controllability (OC) [49,50,51,52]. These properties are binary: a circuit either has sufficient structure and parameters to reach all states or unitaries, or it does not. For the shallow-depth regimes considered here, known results indicate that most circuit families are far from OC and, in some cases, even from PSC.

In contrast, the metrics studied in this manuscript—expressibility based on fidelity distributions [14], majorization of measurement outcome probabilities [13,35], and average entanglement [16,17]—do not address whether full reachability is possible, but instead quantify how uniformly the accessible subset of states is distributed in Hilbert space under random sampling of parameters or gate sequences. As a result, a circuit may be far from PSC or OC while still generating ensembles with near-Haar statistical properties according to these measures. This situation is analogous to space-filling parameterizations, which can yield highly uniform coverage despite low-dimensional structure and unfavorable parameter-space geometry [49,51,52,54]. Consequently, expressibility and majorization probe typical-case, distributional aspects of complexity that are complementary to the worst-case reachability and approximation guarantees provided by dimensional expressivity and controllability theory. Together, these perspectives offer a more complete characterization of the capabilities and limitations of parameterized quantum circuits in the NISQ regime.

## 4. Results

**Figure 2:**
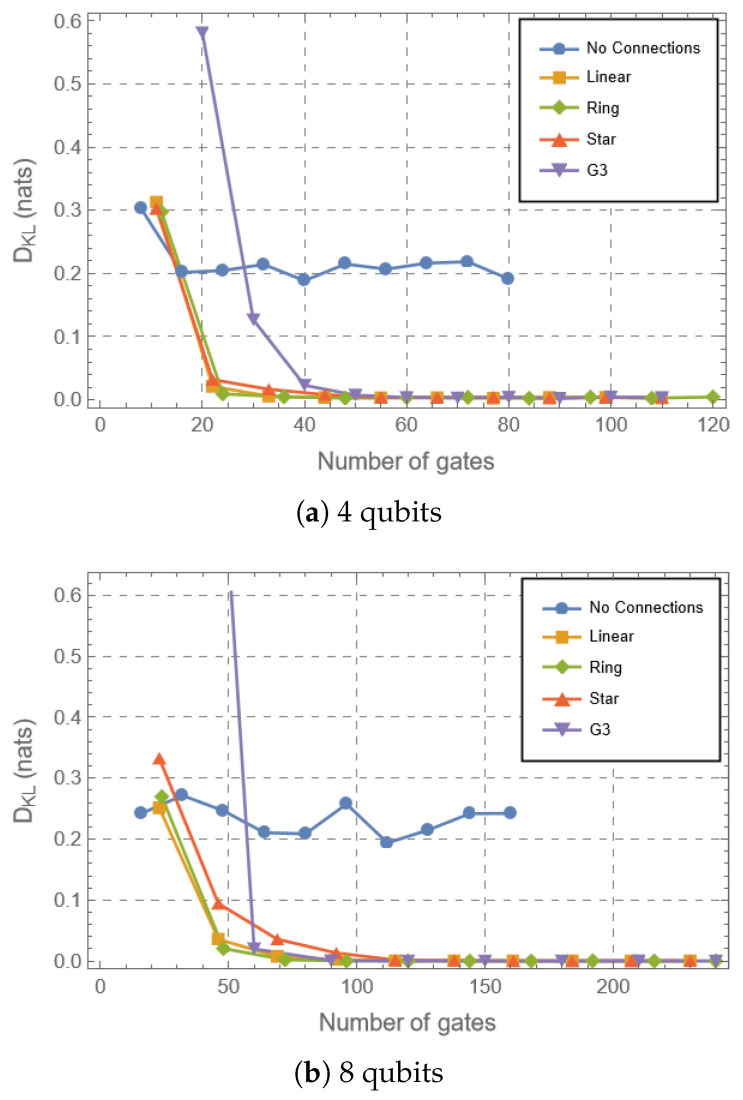
Expressibility for the different circuits considered where the dimensions are n=4,8 qubits, as a function of the number of gates in the circuit.

**Figure 3:**
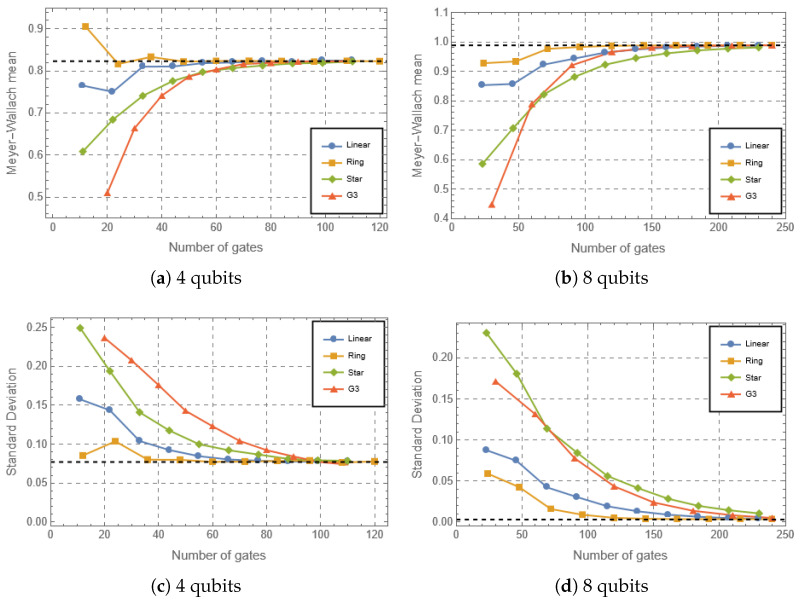
Entanglement and its standard deviation for the different circuits considered for the dimensions of n=4,8 qubits, as a function of the number of gates in the circuit. The only exception not shown is the No Connections circuit, as it does not generate entanglement. The black dashed horizontal lines indicate the average values obtained analytically for the Haar case of both quantities in Refs. [16,17].

We have computed the expressibility, fluctuations of the Lorenz curves and the mean entanglement of all circuit classes for different number of layers/gates applied. Throughout the following discussions, ‘rate of convergence’ refers operationally to the number of gates required for a given metric to approach its Haar value within a fixed tolerance. While we do not extract explicit scaling exponents, relative rates can be inferred from these thresholds. In Figure 2 we can see that the expressibility evolves to its asymptotic value near zero for all circuits with the exception of the no connections topology for the parameterized classes. This is reasonable since in this case there is no possibility of uniformly distributing the states generated given that non-connected qubits can only generate separable states. This decay for all the connected circuits is an expected result for random circuits when composed of universal gate sets for the discrete case and for parameterized quantum circuits in different settings [1,15,25,29,34,35], which converge to an approximation of the Haar measure as a function of the number of gates. The PQCs present a very similar behavior in the lower dimension of 4 qubits (Figure 2a); however, it is possible to notice a hierarchy between the different topologies at the 8-qubit dimension, where the Ring presents the steepest evolution to the Haar case, followed by the Linear, and finally the Star. This behavior can be explained in terms of the average entanglement profiles generated by these circuits. It can be seen in Figure 3 that the Ring circuit generates the highest average entanglement values, followed by Linear, Star, and finally G3, as well as the lowest standard deviations, following a similar hierarchy. Parameterized quantum circuits generating entanglement standard deviations closer to the values observed in the Haar analytical case present a steeper evolution of expressibility, that reflects the convergence to the uniform generation of random states [29]. In the 4-qubit dimension, the standard deviation for the Haar case is higher, so there is more freedom on the values of entanglement that can be generated. By increasing the dimension, the standard deviation decays exponentially, implying restricted values for the entanglement observed in a uniform distribution of random states. This phenomenon is a characteristic of the concentration of measure [55,56]. Therefore, circuits generating entanglement values whose behavior is closer to the uniform random case will present a steeper evolution to the Haar case. These results are more thoroughly discussed in Ref. [29], comparing different parameterized quantum circuits’ architectures together with the topologies. The results here indicate that the same can be observed for random circuits consisting of a few generators that are stochastically sampled and applied to the initial state, where the gates are sampled in the gate set and applied to random qubits as well. We note that while gate count is a standard theoretical metric, practical NISQ implementations must also consider native gate sets, noise properties, gate execution times and error rates, which vary significantly between rotation and CNOT gates across different platforms. Our conclusions therefore pertain to algorithmic complexity growth rather than device-specific performance.

It is relevant to point out in this context that the way the connections between qubits are made and the arrangement of gates are highly influential to the entanglement generation. For instance, it can be the case that probabilistically arranging the gates in the circuit structure (by sampling to which qubit it should be applied) some sets of gates will perform better than others [15,57]. For different classes of random circuits, it can also be the case that sampling random two-qubit gates applied to random neighboring qubits is equivalent to sampling a tensor product between neighbor qubits on even followed by odd sites [58]. The first class consists of sparse random circuits (*local random circuits* in Ref. [58]), while the second is built with a densely connected arrangement of local two-qubit gates (*parallel local random circuit*). In this direction, the circuit structures should be evaluated individually in order to understand the consequences of the choices.

An interesting feature is that the connected parameterized circuits converge faster than the G3 ones to the uniform distribution. In Figure 2, it is possible to see that the limit is reached at approximately half the number of applied gates for the former in the 4-qubit case and appreciable earlier for the 8-qubit case. In this 8-qubit scenario the star topology performs worse than the G3, and it is possible to observe an improvement in the performance of the G3 circuits with a growing number of qubits. These results for the circuit can, again, be explained in terms of the lower standard deviation for the entanglement. Comparing Figure 3c,d, we can see that the standard deviation of the G3 for 8 qubits decays faster at this higher dimension. Furthermore, it is possible to see an inversion between the G3 circuit and the Star circuit for 8 qubits, which is also accompanied by the expressibility behavior.

Finally, it is interesting to compare these results with the rate at which the mean entanglement is generated considering absolute values. The behavior of this measure is qualitatively similar to the expressibility. As a matter of fact, all parameterized circuits outpace G3 ones for the 4-qubit case. The Haar limit is reached at around 50 gates for the Ring and Linear topologies while the Star and G3 reach it at around 80 gates, almost double. Looking at the standard deviation (Figure 3c), we can see that the PQCs will always present smaller standard deviations, closer to the Haar case, than the G3 circuit. In fact, the PQCs present both entanglement values and standard deviations that are closer to the Haar values when compared to G3, from the regime with a small number of gates to the convergence. For the 8-qubit scenario the situation changes in the same way it changed for the expressibility, but slightly better for the G3 circuits, since they reach the Haar limit at approximately 150 gates just after the Linear and Ring parameterized circuits, while the Star topology performs worse, needing about 200 gates for the same result. At this point it is worth noticing that the Star topology makes the entanglement (and general complexity) generation rely heavily on the central qubit; this explains why despite being more connected than a ring or a line, it performs worse in these terms. The standard deviation follows a similar pattern, with the Ring and Linear circuits presenting values closer to the Haar limit from the beginning, and the Star/G3 circuits generating higher values of standard deviations, evolving closer to each other.

**Figure 4:**
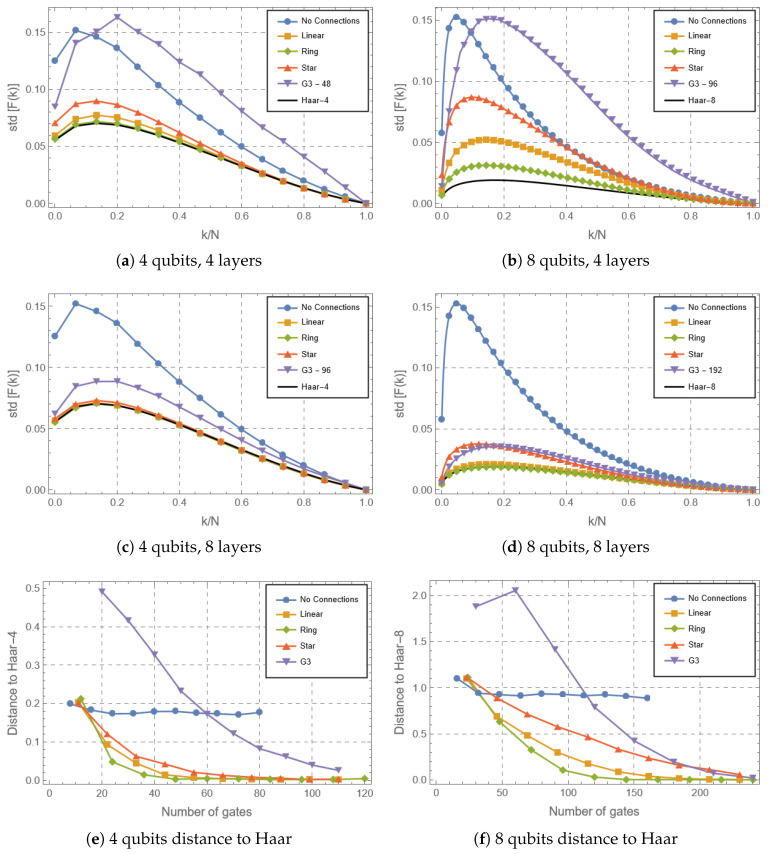
Fluctuations of the Lorenz curves for the different circuits considered and for Haar sampled unitaries of n=4,8 qubits. The number next to G3 indicates the number of gates applied in the random circuit, which is of the same order of the PQCs for comparison. Together with these curves, the distance between the Lorenz curve of each circuit and the appropriate Haar Lorenz curves fluctuations, Equation (7), is also presented.

But, how can we go deeper into the details of the rate at which the states generated reach the optimal complexity? The majorization criterion, based on the fluctuations of the Lorenz curves, provides a complementary point of view since it is related to a more general concept than entropy (this latter being at the foundations of the expressibility measure). Figure 4 presents the behavior of the Lorenz curves for the circuits, together with the corresponding Haar case, for different numbers of qubits and different numbers of layers, together with the quantifier presented in Equation (7). Panels (a) and (b) are the cases of 4 and 8 qubits at 4 layers, and (c) and (d) are the cases of 4 and 8 qubits at 8 layers. Finally, panels (e) and (f) present a quantitative evolution of the comparison between the circuits and Haar curves. In Figure 4a we can see that for the 4-qubit case the parameterized circuits, with the exception of the No Connections scenario (for the same reason stated above), are all near the Haar-4 fluctuations (i.e., the fluctuations corresponding to a uniform sampling over 4 qubits) at 4 layers/48 gates. In fact, the Linear and Ring topologies have almost converged to this result, while the Star-shaped circuits have not. G3’s behavior was closer to the non-connected circuits, and this feature is also verified in the second panel of the first column corresponding to 8 layers/96 gates, where G3 is nearer the Haar-4 results, but still has not converged. It is interesting to highlight that this behavior is slightly different from the one observed for the expressibility: in Figure 2a we can see that G3 has a faster convergence than the No Connections circuit, while the opposite is observed for the distance to Haar-4 considering the Lorenz curves (Equation (7)), presented in Figure 4e in the 4-qubit case. In the former, the G3 behavior is closer to Haar at around 30 gates, while for the latter this is observed only at 60 gates. This can be understood as a slower rate of convergence to the uniformity of the Haar limit that complements the information provided by entanglement focused measures.

On the other hand, it is interesting to consider the interplay between variability (in the sense of freedom to generate local coherences in the No Connections case) and entanglement generation reflected by the quantifiers. In this sense, the variability presented by the random parameters in the No Connections circuit can match in some cases the complexity generated by the entangling G3 circuits, their Lorenz curves being similar when compared to Haar. Therefore, entangling circuits with low variability (G3) will present similar performance to non-entangling circuits with high variability (No Connections), until the entangling low-variability circuits reach an entanglement threshold which starts to dominate the complexity growth. The same behavior is observed in the case of 8 qubits, where the G3 is closer to Haar than No Connections at 60 gates for the expressibility and at 120 gates for the majorization criterion.

As the dimension grows (Figure 4e,f), it is possible to see that a higher number of gates is required to reach the Haar limit. The higher sensitivity to entanglement observed for the expressibility is reaffirmed in this comparison: While the expressibility is capable of detecting that the G3 circuits generate entanglement profile closer to the Haar random case at 8 qubits when compared to the Star case (which can be noticed by the inversion of performance in Figure 2b), the majorization criterion establishes that the G3 will have greater distance to the Haar case until around 160 gates, where both acquire the same performance. Hence, the results here show the contrast in the behavior of different random circuit structures, including continuously parameterized and discrete architectures, at the low-depth regime, and underlines the rich complementarity of the expressibility and majorization criterions for quantifying circuits’ complexity.

## 5. Conclusions

We have found that parameterized quantum circuits, which have a central role for the development of quantum machine learning among other areas, reach maximum complexity with a lower number of layers/gates than a paradigmatic class of random quantum circuits generated with H, T and CNOT gates, the G3. The topologies of the former circuits, i.e., their connectivity between qubits, is crucial, since it is possible to rank their performances based on them. Furthermore, comparing the behavior of two different complexity quantifiers important for Near-Term Quantum Computing, the expressibility and the majorization-based quantifiers, the results provided a way to understand how the performance of parameterized random and discrete random circuits can be compared, with an interesting interplay between entanglement generation, and variability of local gates. Overall, it was possible to observe a better performance for circuits with the Ring and Linear connectivities.

We have used the expressibility and the mean entanglement, which are based on the entropy concept, as measures to characterize such complexity. We have also considered the fluctuations of the Lorenz curves, a criterion based on majorization. The first two led to similar results where the G3 circuits approached the asymptotic values of complexity at a slower pace than the parameterized ones with the exception of the less connected No Connections topology and the Star. Importantly, the expressibility complexity measure was capable of identifying differences in the entanglement behavior of the random circuits, assigning a higher complexity to the entangling G3 circuits when compared to the non-connected topology, as well as presenting sensitivity to changes in the entanglement generation. Moreover, the majorization-based quantifier agreed with the other measures regarding the overall performance, showing that parameterized circuits, in particular the Ring topology, have an excellent performance when compared to G3. Moreover, this measures provides complementary information regarding complexity, showing that the approach to Haar could be slower than that predicted by purely entanglement generation-based quantities. On the other hand, because expressibility, entanglement, and majorization probe different geometric aspects of the state ensemble, they need not induce identical orderings of circuit families. Crossings between curves should therefore be interpreted as metric-dependent rather than absolute indicators of superiority. Showing the interplay between the G3 and the structured circuits, for example, we intend to show the interaction between entanglement/expressibility and variability in circuit structure.

The advantage of random circuits based on PQCs when compared to random circuits consisting of stochastically applied generators is interesting in the near-term quantum computing context. This is owed to the fact that random PQCs have a fixed structure, and even with connectivities that are trivial in quantum computers, e.g., the linear topology, they can present a fast increase in complexity by classically sampling parameters of quantum gates. Therefore, PQCs provide a suitable platform to generate random unitaries in the near term, as random circuits composed of discrete gates, prospected to perform universal computation in the fault-tolerant era, shall require an exponential depth with the dimension to generate a biased replication of the Haar measure for unitaries [1,57]. On top of that, the results in this work provided contrasts and insights into the behavior presented by two different complexity measures, when comparing discrete and parameterized random circuits. It was possible to notice that the increase in complexity is related to an interplay between variability of local quantum gates and entanglement generation, required to achieve highly complex quantum circuits. This discussion highlights the role of the topology of quantum computers, reflected by the connections between qubits.

Intuitively it is reasonable that random PQCs will outperform the discrete gate set G3, as the former has inherently random parameters built in each single-qubit gate. But this is far from obvious given the fact that we are considering shallow circuits and the PQC gates consists of rotations applied to one-qubit subspaces, apart from the CNOT which is common to both kinds of circuits. In this work, we not only confirmed this intuition, but most importantly we provided a quantitative measure of complexity growth. This is made from different viewpoints that notably do not agree in the rate value. Moreover, the results in this work shed light on the role of applying random local parameterized gates to the complexity of quantum circuits, even in the non-entangling context, when compared to these discrete random circuits. Therefore, we expect that this work will provide interesting insight for further investigation on the behavior of random PQCs.

These results pave the way to try and implement these sorts of circuits not only in quantum machine learning architectures, but also in the more specific case of quantum reservoir computing, and to prove quantum supremacy with less resources. For the future we envisage a more realistic evaluation by considering noise, a case which has recently led to a very interesting result in terms of its characterization by means of the spectral properties [59].

## Figures and Tables

**Table 1:** Total number of gates comparing topologies for the circuit structure considered in this work as a function of the number of qubits *n* and number of layers *l*. The number of CNOT gates can be obtained by the calculation of the number of edges appearing in each of the graphs related to the topology. For instance, the Star circuit connects one qubit to every other; therefore, n−1 CNOT gates are required. Each layer of the circuit (denoted *l*) will apply this number of CNOT gates plus 2n parameterized gates.

Topology	Number of CNOTs	Total Number of Gates
No connections	0	(2n)l
Linear	(n−1)l	(3n−1)l
Ring	nl	(3n)l
Star	(n−1)l	(3n−1)l

**Figure A1:**
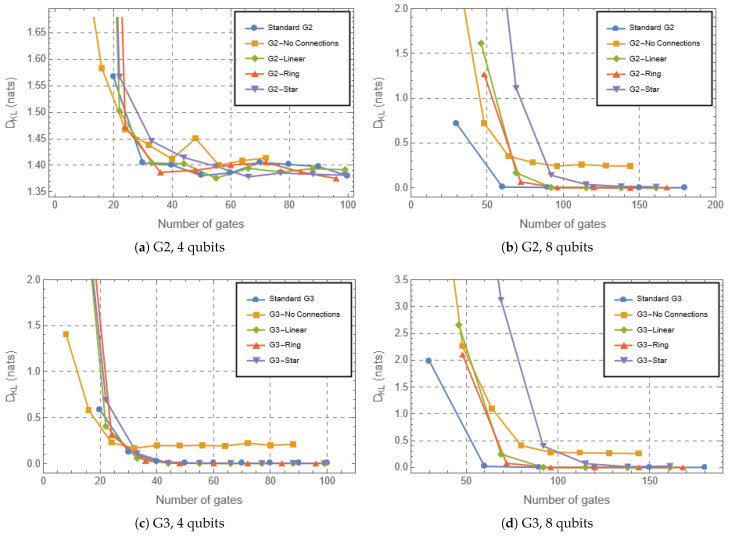
Expressibility for the dense entangling structures applied to G2 and G3 circuits for the dimensions of n=4,8 qubits, as a function of the number of gates in the circuit.

**Figure A2:**
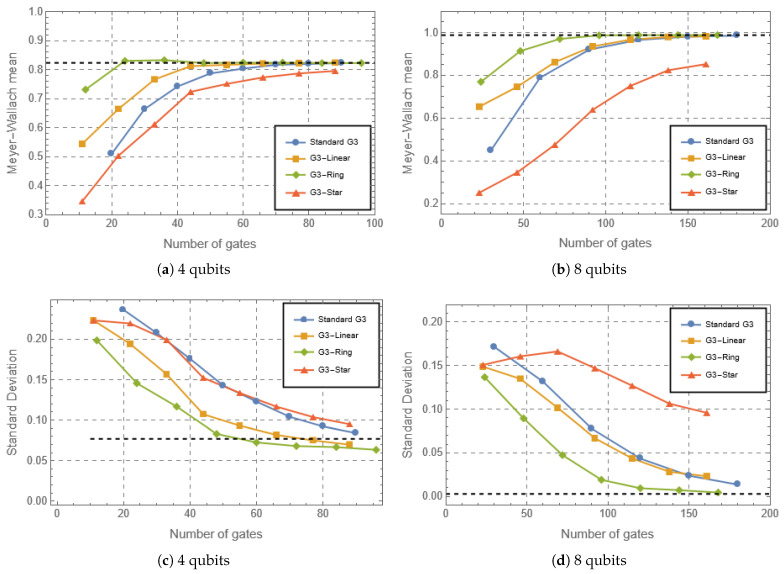
Entanglement and its standard deviation for the dense entangling structures applied to G2 and G3 circuits (exactly same behavior) for the dimensions of n=4,8 qubits, as a function of the number of gates in the circuit. The only exception not shown is the No Connections circuit, as it does not generate entanglement. The black dashed horizontal lines indicate the average values obtained analytically for the Haar case of both quantities in Refs. [16,17].

## Data Availability

All the simulations carried out in this work were made applying the Pennylane Python (version 3.11) Library. Codes and additional results can be checked in the https://github.com/GICorrer/optimal_complexity_of_PQCs (accessed on 5 January 2026).

## Abbreviations

The following abbreviations are used in this manuscript:

NISQ	Near intermediate-scale quantum
PQC	Parameterized quantum circuit
VQA	Variational quantum algorithm

## Appendix A. G2 and G3 Circuits with Dense Entangling Structures

The comparison between the G2 and G3 circuits and the PQCs given different hardware structures might seem unfair at first sight, given that they rely on different types of gates and different entangling structures. The purpose of our analysis in the main sections of this work was to discuss how these differences are important to show that near-term PQCs also provide interesting platforms compared to fault-tolerant based circuits, and how the particularities of each of the complexity quantifiers considered here show that. In this Appendix we present the missing piece of this analysis, which consists in comparing the G2 and G3 circuits in the standard form discussed above with the representatives given the dense entangling structures considered for PQCs. For a fair comparison, we considered these altered circuits consisting of 2n random gates sampled uniformly applied to random qubits, *n* here is the number of qubits, similar to what is done with parameterized gates, followed by the CNOT structures given by the topologies.

First of all, we can see the characteristics of the G2 classes of circuits as a subset of Clifford circuits: its expressibility, as shown in Figure A1, cannot reach values below 1.35 nats for the smaller dimension of 4 qubits. This can be connected to the fact that Clifford circuits are, at most, 2-designs, being able to simulate average values of functions that go up to a second order polynomial in the entries of the random matrices, as the Meyer-Wallach entanglement measure, which can be noticed in Figure A2. For bigger dimensions, the features seem to change, and G2 circuits evolve to smaller values. We attribute this behavior to one of the limitations of the expressibility as a complexity quantifier: It cannot distinguish between design orders, and, for bigger dimensions, this might hinder the possibility of arguing about design orders in terms of general distance to the Haar measure, being specific unitary design quantifiers a more reliable tool. On the other hand, we can see a hierarchy between the Standard G2 circuits and its dense counterparts in the dimension of 8 qubits, where the standard evolves faster to smaller values. The appearance of this distinction for higher dimensions can be connected to the circuit’s structure: the random application of single qubit gates in smaller dimensions might lead to the cancellation of CNOTs from different layers during the coupling part of the circuits, while this is less inclined to happen at larger dimensions.

Regarding the G3 circuits, we notice that they achieve values close to zero for any dimension and topology, excluding the No-Connections for the reason of not generating entanglement. This is associated with the universality of the gate set. As we discussed in the main text, the convergence to the Haar measure of pseudorandom circuits is ultimately dependent on the gates applied and on the connections performed. Panels (c) and (d) in Figure A1 reveal this observation by showing that changing the connectivities, we still obtain a very close behavior for the expressibility of circuits G3 in the Standard, Linear and Ring connectivities, only the Star being slower than the other connected topologies, due to the strictly limited connectivity.

Furthermore, the entanglement evolution shows a relevant distinction between the different topologies applied to G2 and G3, where an hierarchy is established and the topologies present a decreasing convergence speed as Ring, Linear, Standard and Star. Compared to the parameterized circuits, this behavior is still slower, as shown in Figure 3. The distinction between expressibility and entanglement evolution for these G2 and G3 circuits reveals an interesting feature of the interplay between entanglement generation and expressibility of random circuits, which can also be addressed from the viewpoint of non-Cliffordness of random circuits [60]. The interplay between these three quantities, entanglement, magic and complexity as a diagnostic of hardness of classical simulation of a quantum dynamics, are at the core of recent advances in random circuits and many-body dynamics [61,62,63], and are beyond the scope of the present manuscript.
